# Supplementary material for: SOX2-driven enhancer landscape defines the transcriptional architecture of retinogenesis
Source: Development. 2025 Nov 26;152(23):dev204986. doi: 10.1242/dev.204986 (PMC12687333; doi:10.1242/dev.204986)
Supplement: Supplementary information [file develop-152-204986-s1.pdf]

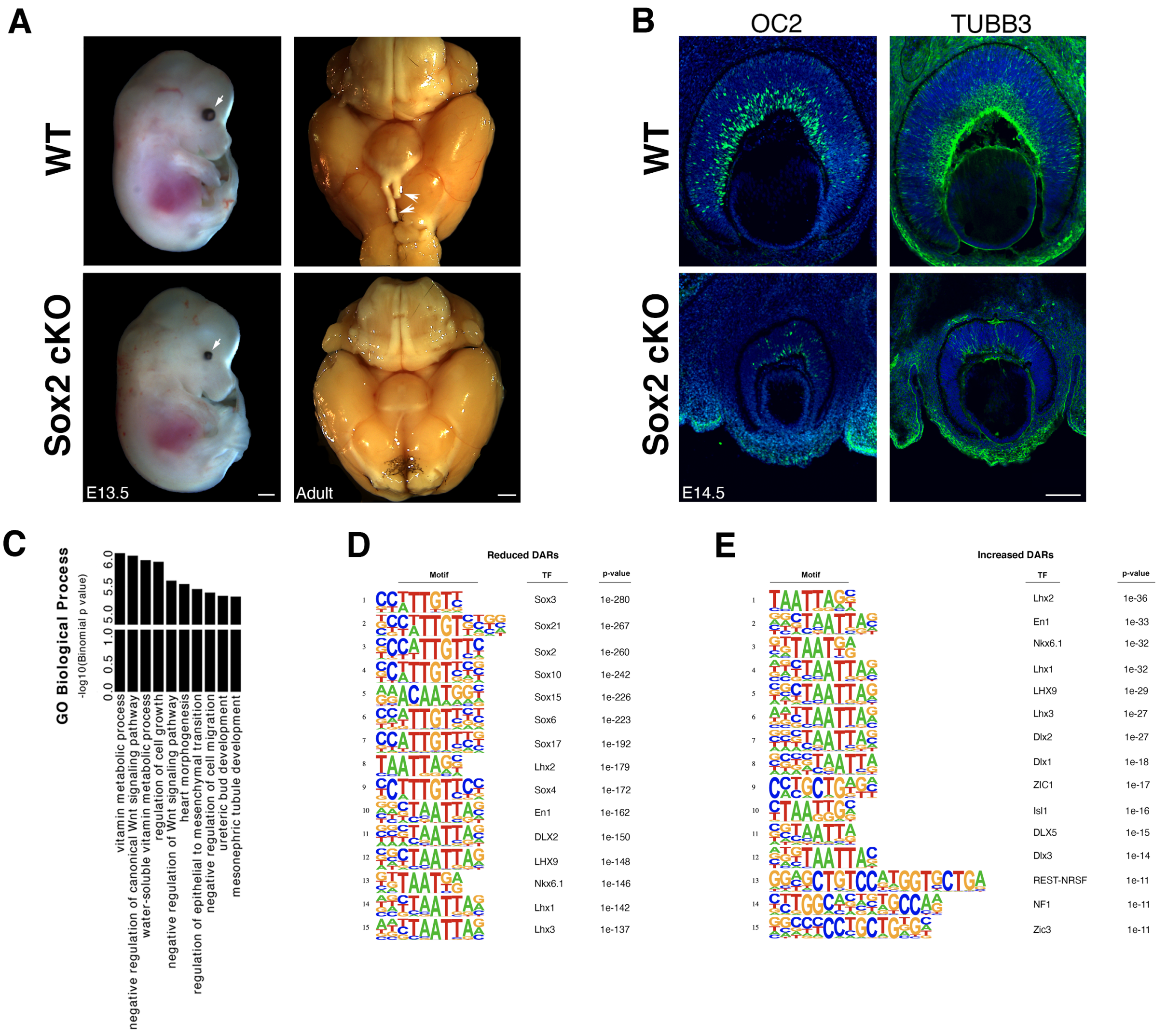

Fig. S1. Loss of SOX2 in RPCs disrupts retinogenesis transcriptional network.

A: Whole mount embryo of *Sox2 CKO* embryos show a smaller eye and loss of optic nerve when compared to control (arrows). B: Representative confocal images showing immunostaining of the RGC markers OC2 and TUBB3 in WT and Sox2 cKO E14.5 retina. C: GO analysis of increased chromatin accessible regions in *Sox2* cKO retina. D-E: Motif analysis of reduced (D) and increased DARs from *Sox2* cKO. For each category, the 15 most enriched binding motifs are shown.

Development • Supplementary information

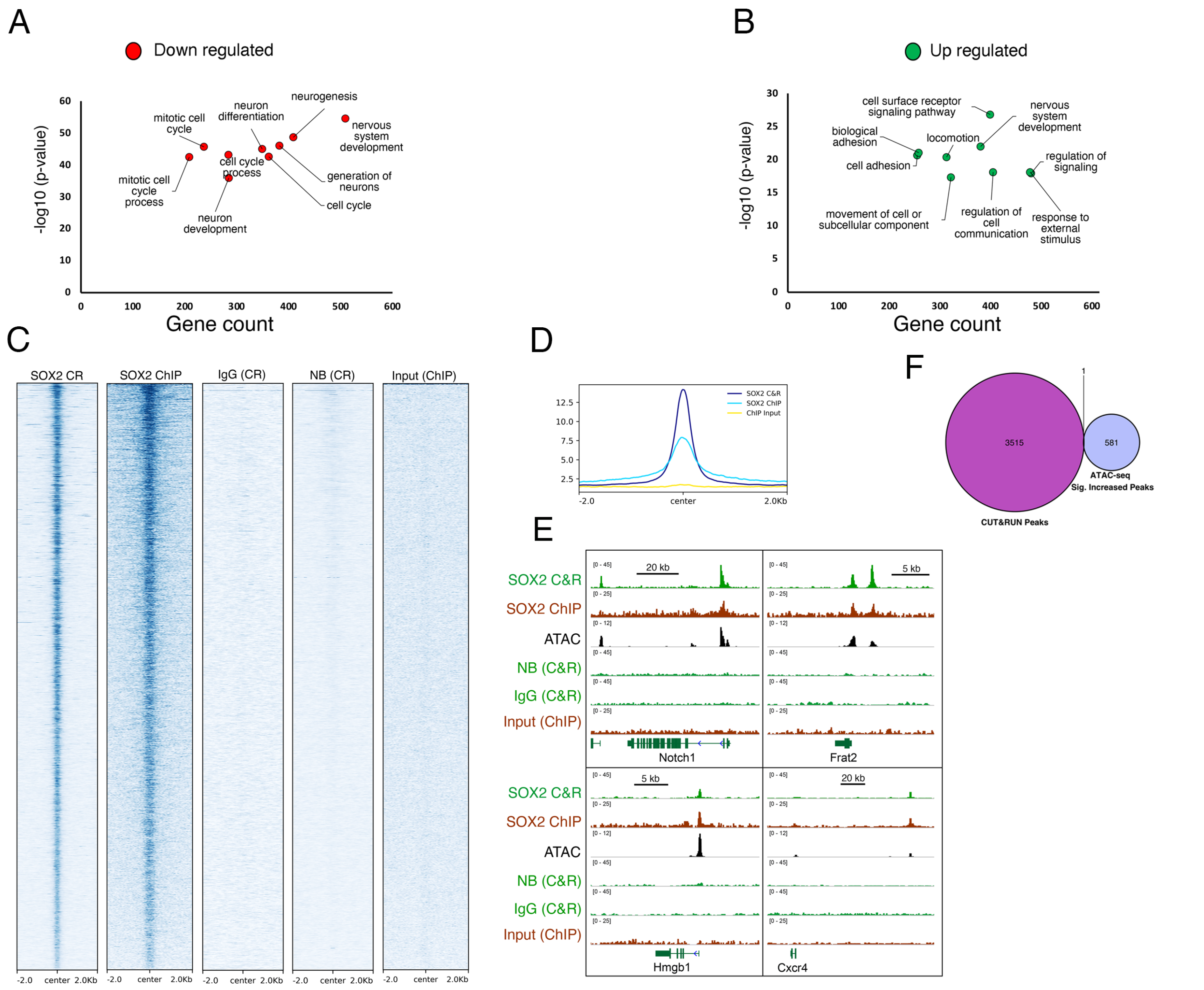

Fig. S2. Analysis of the chromatin landscape in the developing retina upon loss of Sox2.

A-B: GO term analyses of differentially expressed genes from *Sox2* cKO. C: Heat maps of SOX2 binding sites in E14.5 retina as assayed by Cut and Run (SOX2 CR) and ChIP (SOX2 ChIP) with corresponding controls. D: Coverage plots of SOX2 CR, ChIP and control in E14.5 retina. E: Examples of genomic occupancy profiles of SOX2 and controls near the indicated genes showed well-defined peaks of enrichment. F: Venn diagram showing overlap between SOX2 peaks and increased chromatin accessible regions. NB: no antibody

Development • Supplementary information

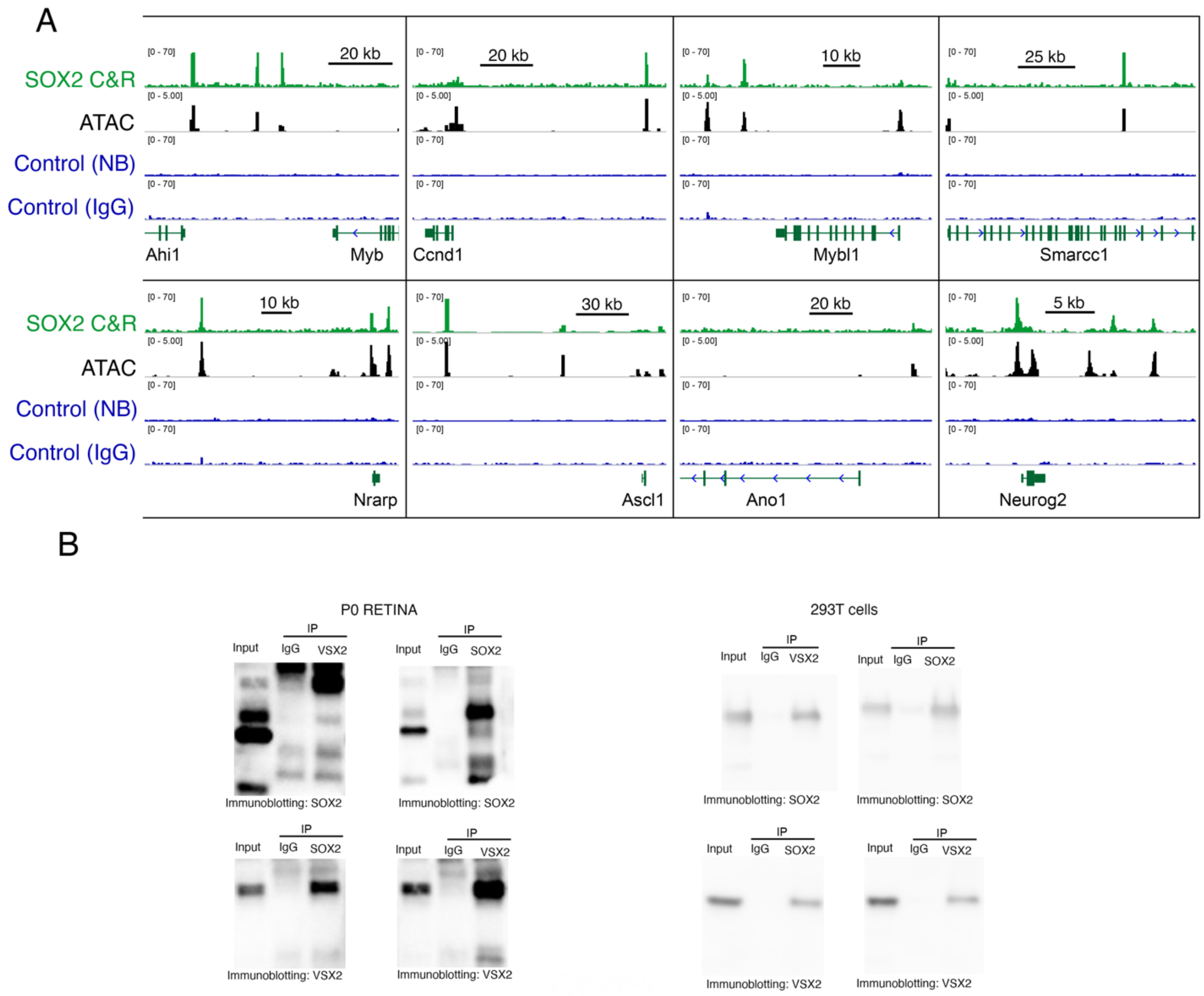

Fig. S3. SOX2 occupies regulatory elements in the developing retina.

A: Genomic tracks of SOX2 occupancy, ATAC and controls nearby indicated genes. B: Extended images of Co-IP data shown in Fig. 5I and J.

Development • Supplementary information

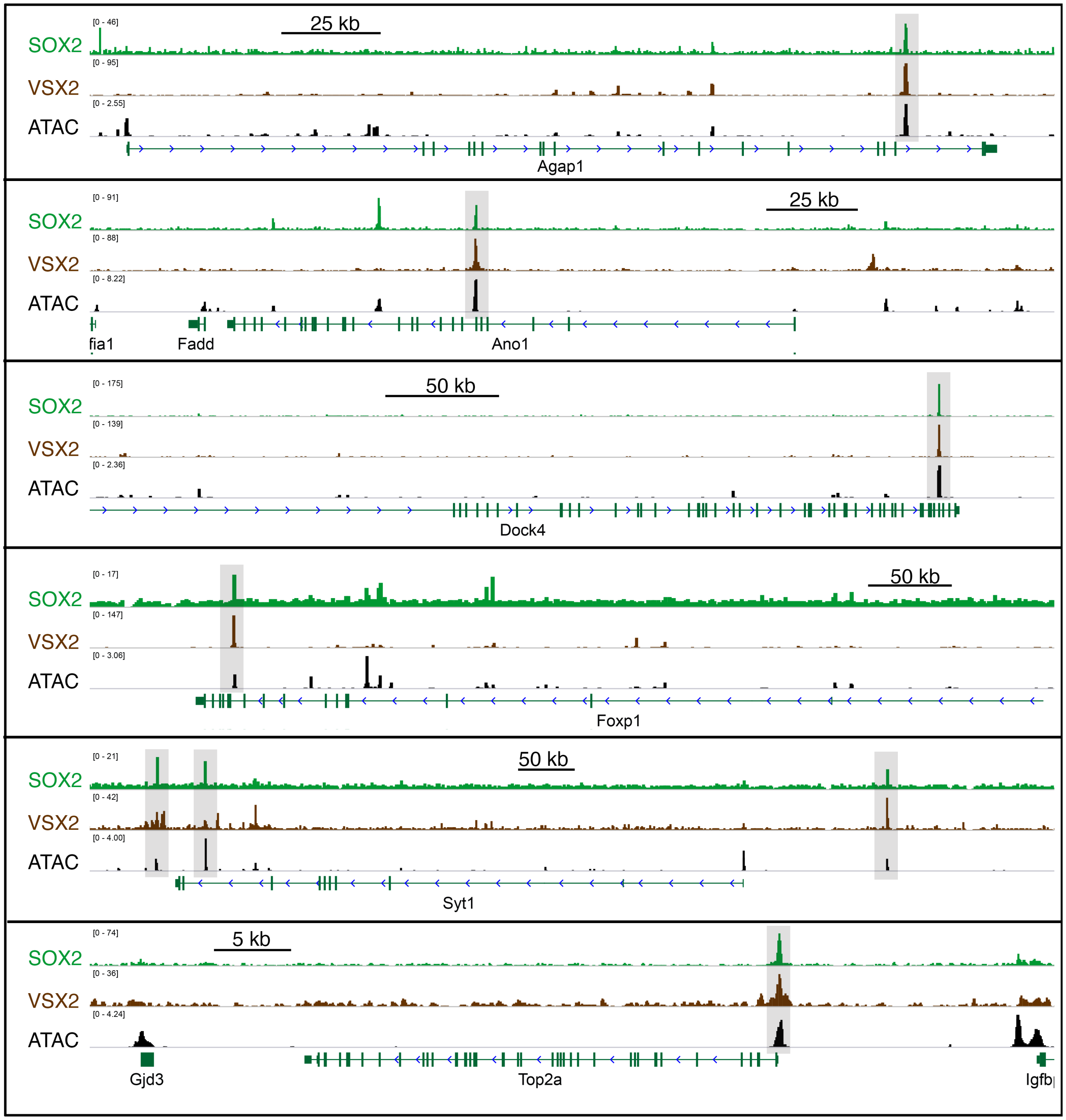

Fig. S4. VSX2-SOX2 co-occupy regulatory elements in the developing retina.

Examples of VSX2-SOX2 co-occupancy (shaded gray areas) in the developing retina at E14.5

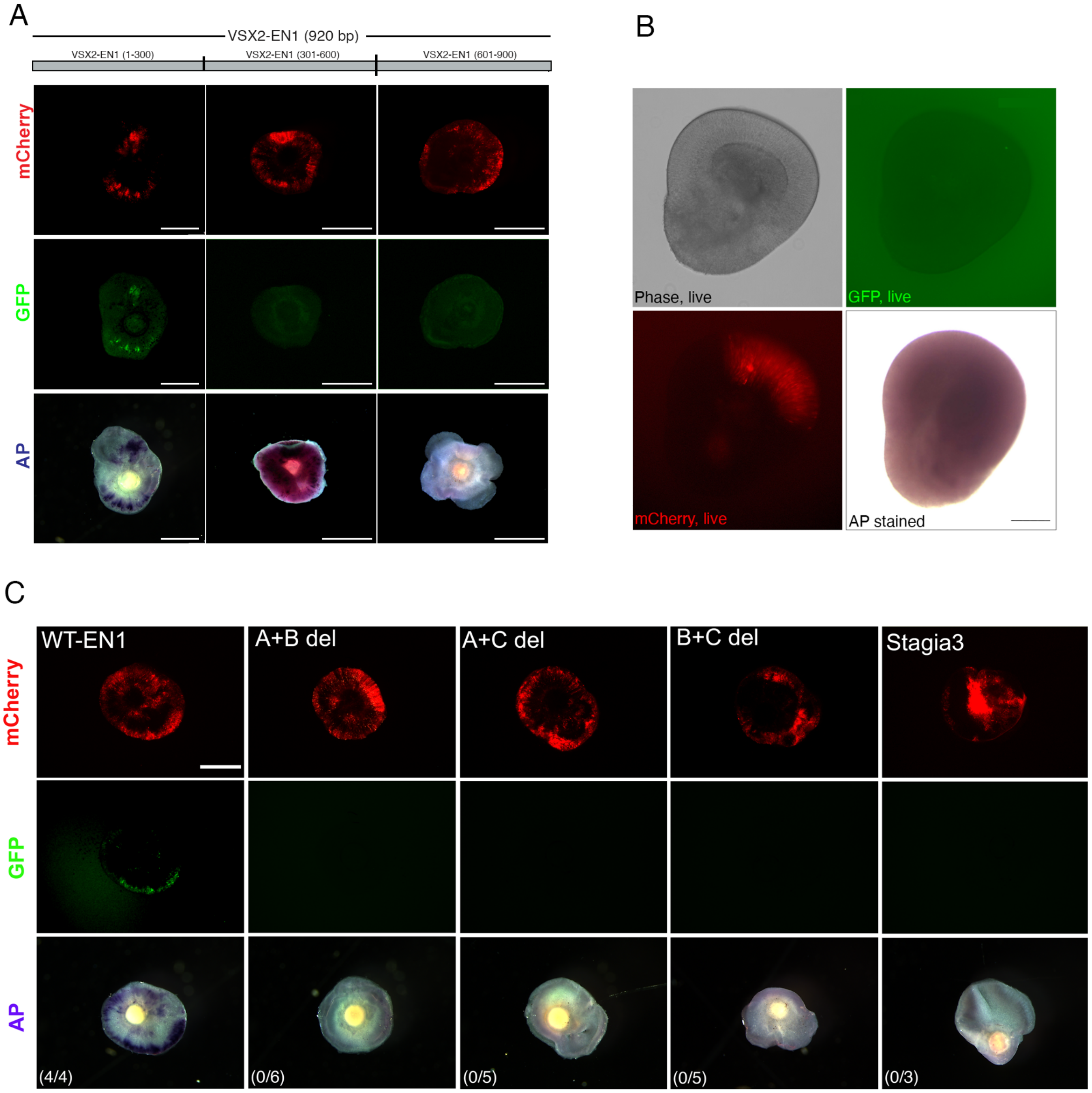

Fig. S5. Functional analysis of Vsx2-EN1 enhancer activities.

A: Functional analysis of VSX2-EN1 regulatory sub-regions. Note that fragment (1-300) maintained robust activities in the retina. scale bar 1000 μm. B: Extended data related to the control presented in Fig. 6B. Shown are the results of electroporation with an empty Stagia3 (control) plasmid in human retinal organoids at Day 44 + 2 DIV. No AP staining or GFP signal was detected. Scale bar 150 μm. C. Mouse retina at day 14.5 + 2 DIV following electroporation with the indicated plasmids. All fragments, except the wild type enhancer, showed a loss of activity, as indicated by the absence of GFP signal and AP staining. scale bar 1000 μm.

Table S1. Differentially accessible regions in Sox2 cKO retina at E14.5

Available for download at
https://journals.biologists.com/dev/article-lookup/doi/10.1242/dev.204986#supplementary-data

Table S2. Differentially expressed genes in Sox2 cKO retina at E14.5

Available for download at
https://journals.biologists.com/dev/article-lookup/doi/10.1242/dev.204986#supplementary-data

Table S3. Annotated peaks for SOX2 Cut and Run at E14.5 Retina.

Available for download at
https://journals.biologists.com/dev/article-lookup/doi/10.1242/dev.204986#supplementary-data

Table S4. Super-enhancer associated genes at E14.5 retina

Available for download at
https://journals.biologists.com/dev/article-lookup/doi/10.1242/dev.204986#supplementary-data

Table S5. Shared SOX2-VSX2 transcriptional and chromatin targets.

Available for download at
https://journals.biologists.com/dev/article-lookup/doi/10.1242/dev.204986#supplementary-data

Table S6. Antibodies used in this study

Antibody	Company	Catalog number	Dilution
SOX2	Active Motif	39844	1:2000
SOX2	R&D	AF2018	1:1000 (3ug in IP)
VSX2	Exalpha	X1180P	1:1000 (3 ug in IP)
VSX2	Santa Cruz	SC-365519X	1:1000
RAX	Takara	M229	1:1000 (immunostaining)
OC2	R&D systems	AF6294	1:300 (immunostaining)
TUBB3	Santa Cruz	Sc-80005	1:300 (immunostaining)
Anti-mouse Alexa-Flour 488	Thermo Fisher Scientific	A32766TR	1:1000 (IHC)
Anti-Sheep Alexa-Flour 488	Thermo Fisher Scientific	A-11015	1:1000 (IHC)
Anti-goat HRP	Jackson ImmunoResearch	705-035-003	1:10K (WB)
Anti-sheep HRP	Jackson ImmunoResearch	313-035-003	1:10K (WB)

Table S7. Genomic coordinates of enhancer segments used in this study

Name	Genomic coordinates (mm10)	**Forward Primer 5'-3'**	**Reverse Primer 5'-3'**
Ascl1+108kb	chr10:87384385-87385402	GCCGAATTCGAACTACACATCACATGCCTC	GCCCTCGAGATGCAGATGGGACAATGACCG
Ccnd1-70kb	chr7:145010752-145011492	GCCGAATTCGGAAGAGATATTCTCAGCTC	GCCCTCGAGGATTGCATGGTGAGGTAGATC
Nrarp-53kb	chr2:25126001-25126633	AATTGAATTCGCAGTACCACGACTCACATAG	ACTCCTCGAGTGGCCTGAAGCCAAACA
Myb+58kb	chr10:21101923-21102580	GCCGAATTCGTGAAGTAGACAGTGTTCATG	GCCGTCGACGGTGATCATGAATCTACACC
Neurog2+9kb	chr3:127642181-127642833	GCCGAATTCCACCCAGTCGTACACAACTC	GCCCTCGAGATGAGTTGTTATCCTCCCGT
Ano1-23kb	chr7:144762313-144763017	AATTGAATTCCTCCAAGTTGGTCCCTTGAATA	ACTCCTCGAGGAAGCCAGTACCGTGAGAAC
Smarcc1+74kb	chr9:110205898-110206471	GCCGAATTCCTCTATAACATCATCCCTGG	GCCCTCGAGTGAACACTGGTTCCACAGAC
Mybl1+51kb	chr1:9648772-9649683	GCCGAATTCCTGGTCCAGATGTACTTGGG	GCCCTCGAGGTAGTGGTGCACACTCCTGG
Mybl1+41kb	chr1:9658349-9659318	GCCGAATTCCCAGAAACGCTTAGCTAGAC	GCCGTCGACGGAAATAGCTTATAGAAGCC
WT-EN1	chr12:84532131-84532430	GCCGAATTCCCTTCCCACCTTTTGTCTGGTA	GCCCTCGAGAGAAATGGACAGAGTGATCCTG
